# Genotyping-by-Sequencing Uncovers the Introgression Alien Segments Associated with Sclerotinia Basal Stalk Rot Resistance from Wild Species—I. *Helianthus argophyllus* and *H. petiolaris*

**DOI:** 10.3389/fgene.2016.00219

**Published:** 2016-12-26

**Authors:** Lili Qi, Yunming Long, Zahirul I. Talukder, Gerald J. Seiler, Charles C. Block, Thomas J. Gulya

**Affiliations:** ^1^Northern Crop Science Laboratory, USDA-Agricultural Research ServiceFargo, ND, USA; ^2^Department of Plant Sciences, North Dakota State UniversityFargo, ND, USA; ^3^Iowa State University Seed Science CenterAmes, IA, USA

**Keywords:** Sclerotinia, basal stalk rot resistance, sunflower wild species, introgression, genotyping-by-sequencing

## Abstract

Basal stalk rot (BSR), caused by *Sclerotinia sclerotiorum*, is a devastating disease in sunflower worldwide. The progress of breeding for Sclerotinia BSR resistance has been hampered due to the lack of effective sources of resistance for cultivated sunflower. Our objective was to transfer BSR resistance from wild annual *Helianthus* species into cultivated sunflower and identify the introgressed alien segments associated with BSR resistance using a genotyping-by-sequencing (GBS) approach. The initial crosses were made between the nuclear male sterile HA 89 with the BSR resistant plants selected from wild *Helianthus argophyllus* and *H. petiolaris* populations in 2009. The selected resistant F_1_ plants were backcrossed to HA 458 and HA 89, respectively. Early generation evaluations of BSR resistance were conducted in the greenhouse, while the BC_2_F_3_ and subsequent generations were evaluated in the inoculated field nurseries. Eight introgression lines; six from *H. argophyllus* (H.arg 1 to H.arg 6), and two from *H. petiolaris* (H.pet 1 and H.pet 2), were selected. These lines consistently showed high levels of BSR resistance across seven environments from 2012 to 2015 in North Dakota and Minnesota, USA. The mean BSR disease incidence (DI) for H.arg 1 to H.arg 6, H.pet 1, and H.pet 2 was 3.0, 3.2, 0.8, 7.2, 7.7, 1.9, 2.5, and 4.4%, compared to a mean DI of 36.1% for Cargill 270 (susceptible hybrid), 31.0% for HA 89 (recurrent parent), 19.5% for HA 441 (resistant inbred), and 11.6% for Croplan 305 (resistant hybrid). Genotyping of the highly BSR resistant introgression lines using GBS revealed the presence of the *H. argophyllus* segments in linkage groups (LGs) 3, 8, 9, 10, and 11 of the sunflower genome, and the *H. petiolaris* segments only in LG8. The shared polymorphic SNP loci in the introgression lines were detected in LGs 8, 9, 10, and 11, indicating the common introgression regions potentially associated with BSR resistance. Additionally, a downy mildew resistance gene, *Pl*_*17*_, derived from one of the parents, HA 458, was integrated into five introgression lines. Germplasms combining resistance to Sclerotinia BSR and downy mildew represent a valuable genetic source for sunflower breeding to combat these two destructive diseases.

## Introduction

Sclerotinia, commonly called “white mold,” is caused by the necrotrophic fungal pathogen *Sclerotinia sclerotiorum* (Lib.) de Bary and is one of the most devastating diseases of sunflower worldwide. *S. sclerotiorum* infects all parts of the sunflower plant and causes three distinct diseases: basal stalk rot (BSR), mid-stalk rot (MSR), and head rot (HR). BSR is dependent on the infection of roots with mycelia, while MSR depends on ascospores infection of the leaves and stem, and HR depends on ascospores infecting the capitulum (Gulya et al., 1997). The MSR is not as common as the BSR or HR in the United States. The latter two diseases cause serious losses of yield and seed quality during epidemic years under appropriate environmental conditions. Field evaluation of germplasm for resistance to Sclerotinia BSR and HR revealed that there was no correlation between the two, suggesting a different inheritance of resistance to Sclerotinia in sunflower (Gulya et al., 1989; Talukder et al., 2014).

Sclerotinia BSR is historically the most widespread and economically serious disease of sunflower in the United States. Sclerotinia overwinters as sclerotia in the soil and in plant debris. Sunflower infection occurs through the roots any time sunflower is planted in a sclerotia infested field. As the infection spreads, a girdling, basal stem canker is formed with a tan to manila basal lesion with white mycelium or black sclerotia at the soil line, causing the plant to wilt and die. Thus, a 100% yield loss of each systemically infected plant is expected (Gulya et al., 1997). In addition, infection leads to increased levels of sclerotia in the soil, which results in having to rotate away from sunflower production for several years in that field. There are no efficient chemical controls available for this pathogen; thus, breeding resistant sunflower lines and hybrids is considered the most effective management tactic for BSR in sunflower. No complete resistance has been identified in cultivated sunflower or in its wild relatives. However, quantitative genetic variations among some varieties have been described (Tourvieille et al., 1996; Degener et al., 1998, 1999; Gulya et al., 2010; Talukder et al., 2014). Previous efforts to characterize BSR resistance indicated that the trait is quantitatively inherited for which the majority of the genetic variation is due to additive gene effects (Mestries et al., 1998; Van Becelaere and Miller, 2004; Talukder et al., 2016). Thus, breeding sunflower for Sclerotinia BSR resistance has relied on the introgression of genetic factors from various partially resistant accessions using recurrent selection or pedigree breeding methods (Miller and Gulya, 1999, 2006). Improved Sclerotinia resistance and selection efficiency should be achieved by pyramiding the various resistance genes using marker-assisted selection. However, progress in breeding sunflower with BSR resistance is hampered due to the lack of effective sources of resistance in cultivated sunflower and closely linked molecular markers. The incorporation and molecular mapping of major QTL resistance from diverse genotypes are still urgently needed.

Wild sunflower species and the sunflower crop are native to North America (Harter et al., 2004). *Helianthus* comprises 53 wild species, 14 annual, and 39 perennial (Schilling, 2006; Stebbins et al., 2013). Several of these species are described as potential sources of resistance to *S. sclerotiorum* (Henn et al., 1997; Cerboncini et al., 2002; Rönicke et al., 2004; Jan and Seiler, 2008; Seiler, 2010) and, thus, can be used to mine resistance genes and broaden the genetic diversity of Sclerotinia resistance. Introgression from wild annual *Helianthus* species is more likely to be successful because the species are diploid with a basic chromosome number of *n* = 17, same as cultivated sunflower. Thus, it is easier to cross the species with the cultivated sunflower than the perennial species, and meiotic recombination can be achieved through homoeologous pairing in the progenies. In a project funded by the USDA National Sclerotinia Initiative, a wide array of 460 accessions of 14 wild annual sunflower species were evaluated for Sclerotinia BSR resistance over 3 years (2007–2009) under intensive disease pressure in the greenhouse. Accessions exhibiting partial resistance to BSR were identified in the wild annual species *H. argophyllus, H. debilis, H. praecox*, and *H. petiolaris* (Block and Gulya, 2008; Block et al., 2009, 2010).

*H. argophyllus* is a wild annual species that is mainly distributed in the sandy coastal plains of southern Texas (Rogers et al., 1982). It has been a valuable source of disease resistance genes for rust, downy mildew, and Sclerotinia (Miller and Gulya, 1988; Seiler, 1991, 2010; Gulya, 2005; Qi et al., 2016). Scientific attention has been given to *H. petiolaris* as the first cytoplasmic male-sterility (PET1 CMS) was discovered in this species and bred into cultivated sunflower (Leclercq, 1969). Despite the large number of CMS sources available in sunflower, only the PET1 CMS has been exclusively used for commercial hybrid seed production (Serieys, 2005). To exploit the potential resistance present in undomesticated crop wild relatives, the resistance must first be transferred into cultivated sunflower to facilitate field testing. Both *H. argophyllus* and *H. petiolaris* belong to the secondary gene pool of sunflower and are closely related to common sunflower *H. annuus*, the donor of the cultivated sunflower (Burke et al., 2004; Heesacker et al., 2009; Kantar et al., 2015). Thus, the currently available genomic resources can be used to monitor alien introgressions from the donor species in the cultivated sunflower backgrounds. Due to quantitative nature of BSR resistance, a large number of markers are required to uncover the introgressed alien fragments associated with BSR resistance throughout the sunflower genome.

High-throughput next-generation sequencing (NGS) technology has led to remarkable advances in whole genome sequencing. Genotyping-by-sequencing (GBS) is a novel application of NGS protocols for discovering and genotyping SNPs in crop genomes and populations (Elshire et al., 2011; Poland and Rife, 2012). As a cost-effective, high-throughput, and unique tool for genomics-assisted breeding, GBS is particularly powerful for the detection of alien chromosomal segments, which associate with quantitative traits introduced into the breeding pool (Arbelaez et al., 2015). Here, we report the transfer of Sclerotinia BSR resistance from the wild annual species of *H. argophyllus* and *H. petiolaris* into cultivated sunflower, the development of high levels of BSR resistant introgression lines, and the identification of the introgressed alien segments associated with BSR resistance using the GBS approach.

Downy mildew (DM), caused by *Plasmopara halstedii* (Farl.) Berl. et de Toni, is another serious sunflower disease globally. Unlike Sclerotinia, a single gene controls resistance to downy mildew. In this study, we integrated the broad-spectrum downy mildew resistance gene, *Pl*_17_, derived from HA 458 into BSR resistant lines, providing breeders with germplasm resistant to the two of the more serious sunflower diseases.

## Materials and methods

### Plant materials

Four accessions of *H. argophyllus* (PI 435623, PI 494573, PI 649863, and PI 649864) and six accessions of *H. petiolaris* (PI 435815, PI 435843, PI 468811, PI 468818, PI 451978, and PI 549165) were selected as BSR resistant donors identified by Block and Gulya (2008), Block et al. (2009, 2010). All four *H. argophyllus* accessions were collected from Texas. Of the six *H. petiolaris* accessions, four accessions (PI 435815, PI 435843, PI 468811, and PI 468818) were sub-species *fallax*, with the former three collected in New Mexico, while the latter one was collected in Arizona. The remaining two *H. petiolaris* accessions, PI 451978 and PI 549165 were collected from Kansas and South Dakota, respectively. The cultivated sunflower parents included three inbred lines, nuclear male sterile (NMS) HA 89 (PI 559477), HA 89 (PI 599773) with normal cytoplasm, and HA 458 (PI 655009). HA 89 is an inbred maintainer line released by USDA-ARS and the Texas Agricultural Experiment Station in 1971. The NMS HA 89 was induced by streptomycin treatment of HA 89 that possessed a single recessive nuclear male sterility gene *ms9*, released by the USDA-ARS and the North Dakota Agricultural Experiment Station, Fargo, ND in 1990 (Jan and Rutger, 1988; Chen et al., 2006). HA 458 is a high oleic and downy mildew resistant germplasm carrying the *Pl*_*17*_ gene released by USDA-ARS and the North Dakota Agricultural Experiment Station, Fargo, ND in 2010 (Hulke et al., 2010; Qi et al., 2015). Both HA 89 and HA 458 are susceptible to BSR disease. The commercial oil-type hybrid Cargill 270 was used as a susceptible check, while the Croplan 305 hybrid and an inbred line HA 441 were used as resistant controls in this study.

### Development of introgression lines by backcross and selection

**Figure 1:**
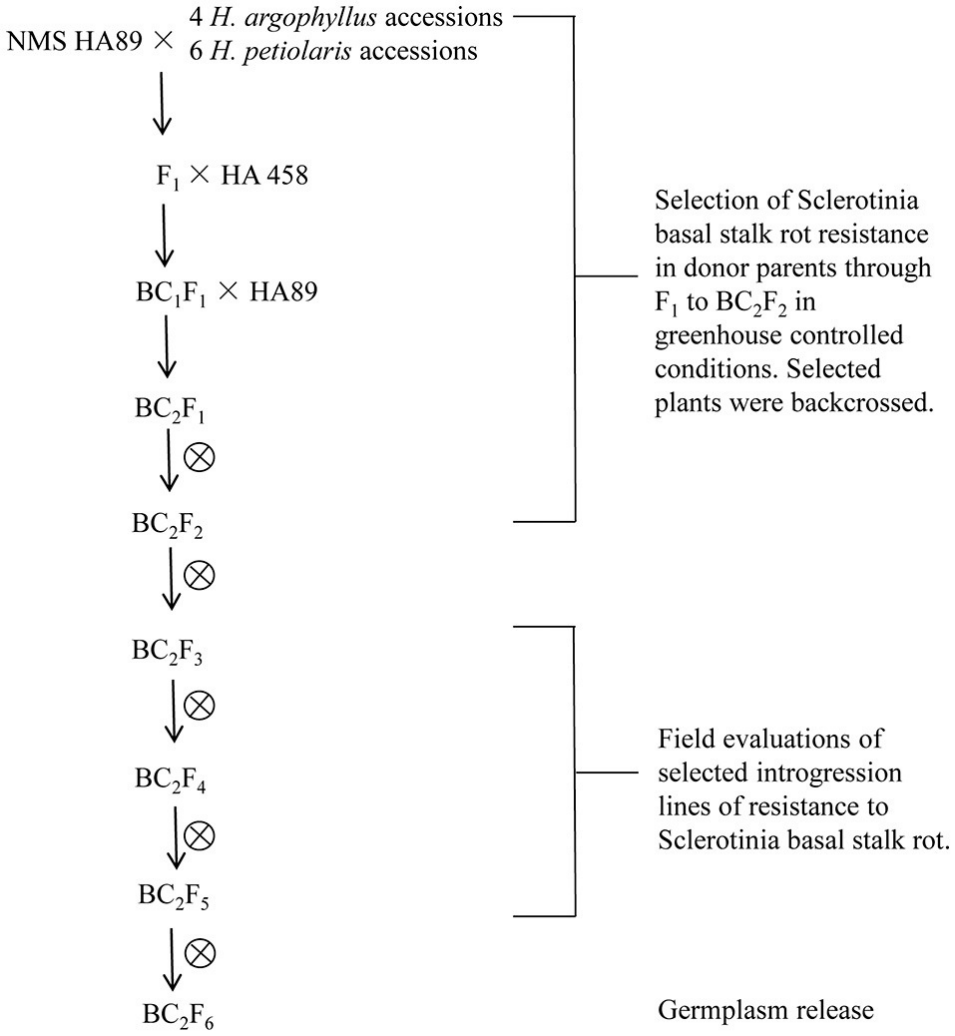
**Schematic diagram of the Sclerotinia resistant germplasm selection from the wild species crosses**.

Due to the open-pollinated nature of wild *Helianthus* species populations, they are segregating for disease resistance. The BSR resistant plants were selected from 10 accessions of the two wild species, *H. argophyllus* and *H. petiolaris* grown in the greenhouse. To promote early flowering of the *H. argophyllus* accessions, 5-week old greenhouse grown seedlings were transferred to a growth chamber to increase their dark period from 9 to 16 h (25/20°C, 8/16 h light/dark cycles). After 1 month, the treated plants were returned to greenhouse. Crosses were made in greenhouse in 2009. To eliminate laborious emasculation process, NMS HA 89 was initially used as the female parent in crosses with the wild species. One to three thousands florets of NMS HA 89 were pollinated by *H. argophyllus* and *H. petiolaris* pollen in each hybridization. Phenotypic selection of BSR resistant F_1_s and successive generations through BC_2_F_2_ were performed from 2010 to 2012 in the greenhouse under controlled conditions (Figure 1). The selected resistant F_1_s were crossed to HA 458, and the derived hybrids from this cross were treated as BC_1_s. The selected BC_1_s were crossed to HA 89 again. The BC_2_F_1_ plants were self-pollinated and advanced to BC_2_F_2_ generation in the greenhouse, followed by continuous self-pollination for four generations. The BC_2_F_3_ families and the progenies of the following generations were grown in the field during 2012–2015 to obtain seeds for the field experiments.

### Inoculum production and greenhouse inoculation

**Figure 2:**
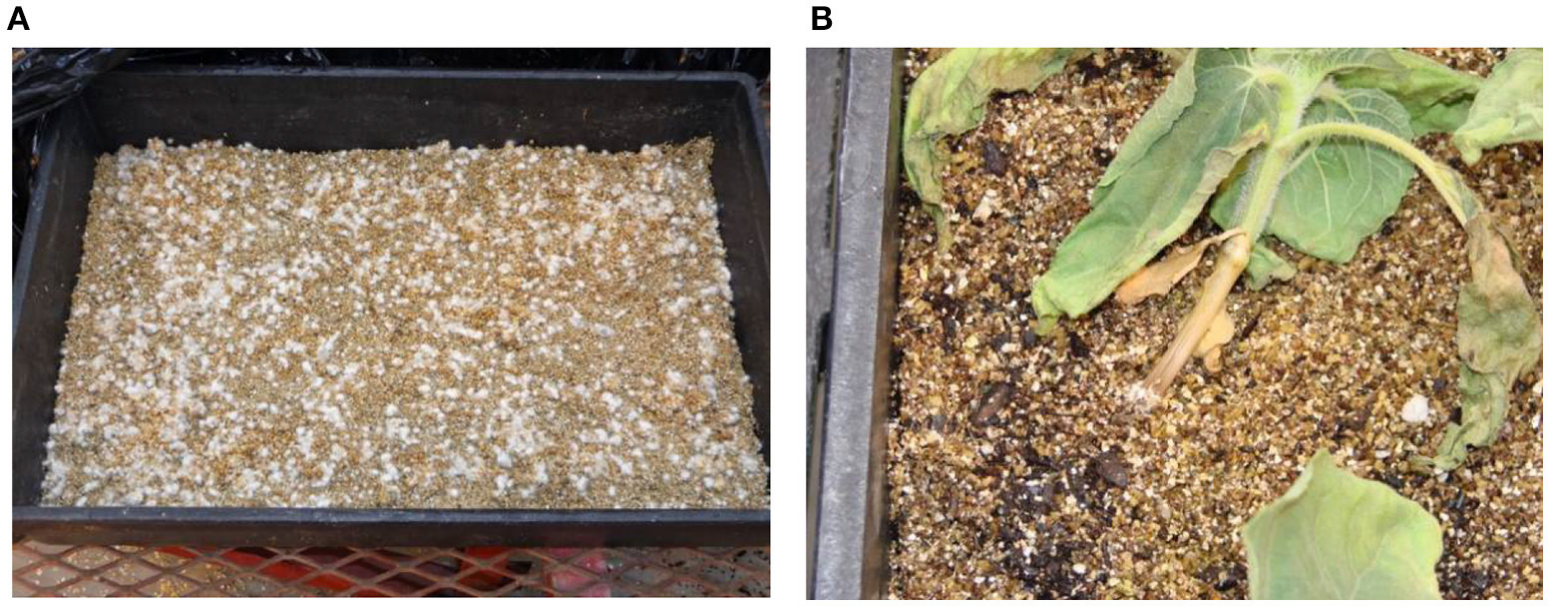
**Greenhouse inoculation of the Sclerotinia basal stalk rot. (A)** An inoculation tray filled with mycelium-bearing millet seeds (120 g). **(B)** The susceptible check Cargill 270 shows disease symptoms 7 days after inoculation.

The inoculums for the greenhouse screening trials were made using the *S. sclerotiorum* fungal isolate, NEB-274. The inoculum was produced by growing the fungus on autoclaved white Proso millet (*Panicum miliaceum* L.) for 7–9 days (before any sclerotia develop), drying the inoculum to 10% moisture, and storing it at 4°C until needed. The infected millet seeds were spread on an inoculation tray (120 g of inoculated millet for one 54.6 × 34.3 × 10.2 cm tray), containing a layer of vermiculite with a fiberglass screen on the bottom (Figure 2). The inoculated trays were placed in a growth chamber at ~22°C under moist conditions in the dark for 3 days and were then transferred to the greenhouse. The seeds were planted in 24-cell plastic flats (each cell 5.7 × 7.6 cm) filled with Sunshine SB 100B potting mixture, and 3-week-old sunflower seedlings were removed from the plastic flats and were placed on the top of the inoculated millet in the tray (24 plants per tray). The base of the seedlings was then covered with a layer of vermiculite to provide sufficient moisture when irrigated with water and incubated in the greenhouse at soil temperatures from 22 to 24°C. The plants were observed daily for disease development. Susceptibility to Sclerotinia BSR was measured as disease incidence (DI), which was scored as a percentage of dead and/or wilted plants at 14–18 days after inoculation.

### Field experiments

The selected BC_2_F_3_ families and their progenies of BC_2_F_4_ and BC_2_F_5_, along with the recurrent parent and susceptible and resistant checks, were evaluated in inoculated field nurseries for their reaction to BSR at seven environments (locations and /or year) in North Dakota and Minnesota during 2012–2015. The field trials were performed with a randomized complete block design with two replications in 2012 and 2013, and three replications in 2014 and 2015. The plots were a 6-m long single row with 0.75 m spacing between the rows. Twenty-five seeds were planted per row and later thinned to 20 plants. Field nurseries were artificially inoculation following the method developed by Gulya et al. (2008) for large-scale field evaluation for Sclerotinia BSR resistance. Ninety-grams of Sclerotinia mycelia of the isolate NEB-274, grown on millet seeds were placed in furrows 10 cm from the row at a depth of 5 cm at the V6 growth stage (Gulya et al., 2008). The susceptibility to BSR was measured by disease incidence (DI) at maturity in the field, which is expressed as the percentage of infected plants showing BSR lesions in each row.

### Genotyping-by-sequencing and SNP calling

GBS was performed in the parental lines, HA 89 and HA 458, and the selected introgression lines to track the wild *Helianthus* segments associated with BSR resistance. Due to highly heterozygous nature of the BSR resistance donors, *H. argophyllus* and *H. petiolaris* accessions were not included in the GBS. Young leaf tissue was collected and lyophilized from the selected sunflower lines. Genomic DNA was extracted from the lyophilized tissues using the DNeasy 96 plant kit following the manufacturer's instructions (Qiagen, Valencia, CA, USA), and the quantity and quality of DNA were determined using a NanoDrop 2000 Spectrophotometer (Thermo Fisher Scientific, Wilmington, DE, USA). The GBS was performed following the protocol described by Elshire et al. (2011). Approximately, 2.0 μg of genomic DNA was sent to the Institute for Genomic Diversity at Cornell University for GBS (http://www.biotech.cornell.edu/brc/genomic-diversity-facility/services). GBS libraries were constructed using the restriction enzyme *Eco*T221. For SNP calling, the sequence tags were aligned to the draft sunflower reference genome HA412.v1.0. (https://www.sunflowergenome.org/genomeassembly.html). The SNPs were named with a prefix of S1 to S17, which corresponds to the 17 sunflower linkage groups (LGs), followed by a number representing the physical position of the SNP on the genome. The SNPs that were not able to be assigned to one of the 17 LGs had the prefix S18.

### Downy mildew tests and marker genotyping of the downy mildew *R*-gene *Pl_*17*_*

The introgression lines of the BC_2_F_5_, along with the parental lines HA 89 and HA 458 (carrying *Pl*_*17*_), were screened for resistance to downy mildew using the North America (NA) downy mildew race 734, a virulent race identified in USA in 2010 (Gulya et al., 2011). The whole seedling immersion method was used for the seedling tests as described by Gulya et al. (1991) and Qi et al. (2015). The susceptible plants showed an abundant white sporulation on the underside of the cotyledons and true leaves, while the resistant plants had no sporulation.

Simple sequence repeat (SSR) marker ORS963 and two single nucleotide polymorphism (SNP) markers, SFW04052 and SFW08268 that are linked to the *Pl*_*17*_ downy mildew resistance gene were used to screen the introgression lines (Qi et al., 2015). Polymerase chain reaction (PCR) for SSR primers was performed on a Peltier thermocycler (Bio-Rad Lab, Hercules, CA, USA) with a touchdown program as described by Qi et al. (2011). Genotyping of the SNPs was performed using a newly developed technique of converting the SNPs into length polymorphism markers described by Qi et al. (2015). The PCR products were diluted 40–60 times and size segregated using an IR^2^ 4300/4200 DNA Analyzer with denaturing polyacrylamide gel electrophoresis (LI-COR, Lincoln, NE, USA).

### Statistical analysis

An analysis of variance (ANOVA) was performed on the BSR DI data obtained from the replicated field screening trials of the BC_2_F_3_ and the subsequent generations using the GLM procedure of SAS version 9.3 (SAS Institute, 2011). The least significant difference (LSD) test was used to compare the DI means among the introgression lines at the 5% level of significance (Steel and Torrie, 1980).

## Results

### Wild hybridization and introgression of sclerotinia BSR resistance

The F_1_ seed set ranged from 0 to 1.89% for the four *H. argophyllus* accessions, and 0.13–13.53% for the six accessions of *H. petiolaris* (Table 1). Among the four accessions of *H. argophyllus*, only PI 494573 produced 45 F_1_ seeds from the 2375 pollinated florets. Among the six *H. petiolaris* accessions, PI 435815 had the highest seed set of 13.53%.

**Table 1:** **F_1_ hybrid seed set from the crosses of NMS HA 89 with the selected basal stalk rot resistant plants from wild sunflower accessions of the *H. argophyllus* and *H. petiolaris***.

**Crosses**	**No. of florets pollinated**	**No. of seeds obtained**	**Seed set (%)**
NMS HA89 × *H. argophyllus* PI 435623	2048	1	0.05
NMS HA89 × *H. argophyllus* PI 494573	2375	45	1.89
NMS HA89 × *H. argophyllus* PI 649863	1462	0	0.00
NMS HA89 × *H. argophyllus* PI 649864	1153	0	0.00
NMS HA89 × *H. petiolaris* ssp. *fallax* PI 435815	2468	334	13.53
NMS HA89 × *H. petiolaris* ssp. *fallax* PI 435843	3062	61	1.99
NMS HA89 × *H. petiolaris* ssp. *fallax* PI 468811	1016	96	9.45
NMS HA89 × *H. petiolaris* ssp. *fallax* PI 468818	1002	2	0.20
NMS HA89 × *H. petiolaris* PI 451978	3629	173	10.62
NMS HA89 × *H. petiolaris* PI 549165	2342	4	0.13

**Figure 3:**
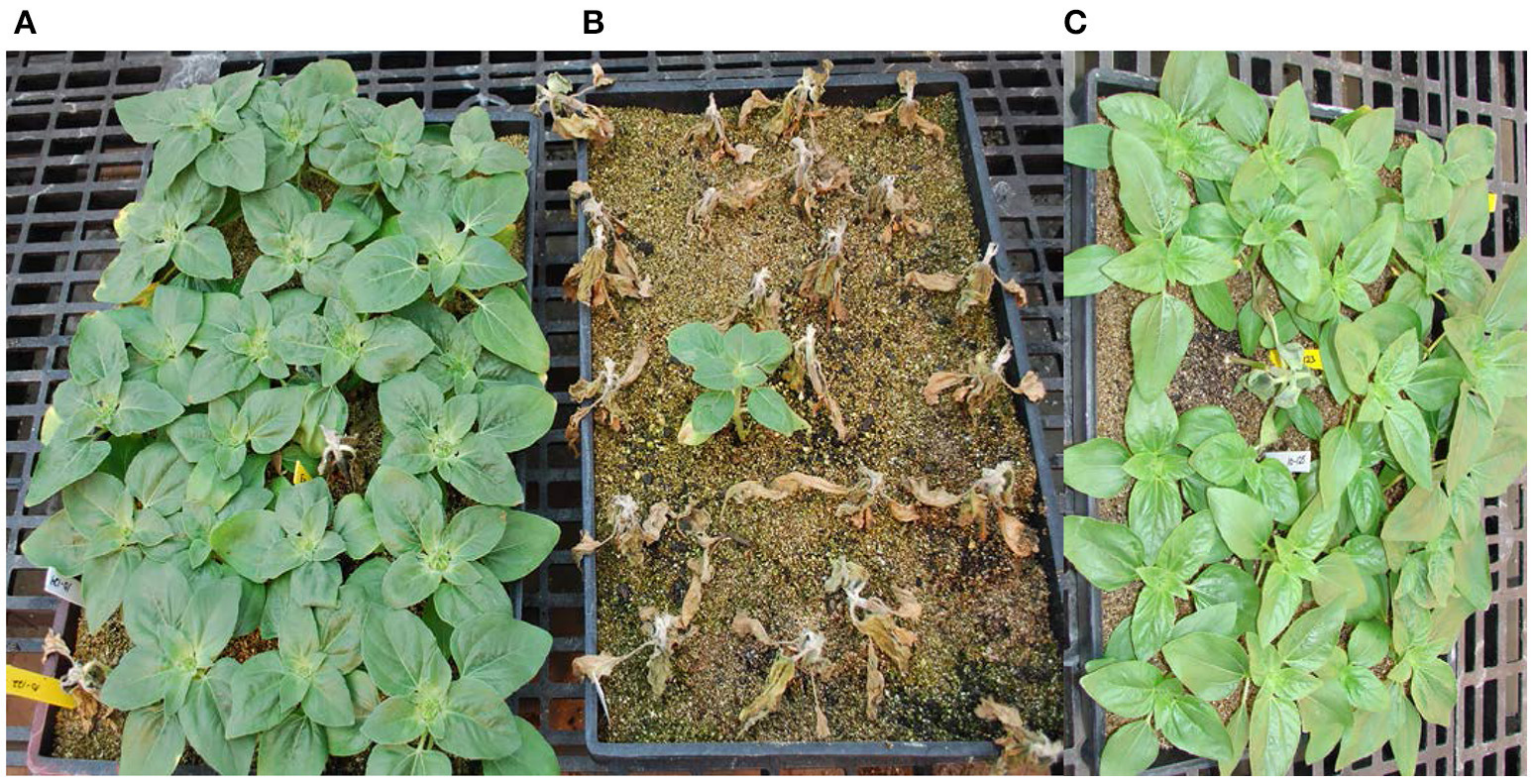
**Sclerotinia basal stalk rot evaluations of the F_1_ hybrids. (A)** The F_1_ hybrids of NMS HA 89/*H. argophyllus* PI 494573 scored 18 days after inoculation. **(B)** Susceptible check Cargill 270. **(C)** The F_1_ hybrids of NMS HA 89/*H. petiolaris* PI 435843. Dead plants with yellow labels in **(A,C)** are susceptible checks.

The hybrids of the *H. argophyllus* accession PI 494573 and the four *H. petiolaris* accessions, PI 435815, PI 435843, PI 451978, and PI 468811, with enough F_1_ seeds were tested for BSR resistance. All of the F_1_s showed a high level of resistance. The DI was significantly lower in the F_1_ hybrids than in the susceptible checks and the recurrent parent HA 89 (Table 2, Figure 3). The *H. argophyllus* F_1_s had a DI of 4.5% compared to 14.0 and 18.0% for resistant checks HA 441 and Croplan 305, respectively. The four *H. petiolaris* F_1_ hybrids had a DI that ranged from 2.0 to 11.0%, which was also lower than both of the resistant checks. The results indicated that the BSR resistance was transferred from the wild *Helianthus* species into the cultivated sunflower background and was expressed in the hybrids. The resistant F_1_ plants from five crossing combinations were used as male parents in crosses to HA 458. Only two F_1_s produced BC_1_ seeds, one each for *H. argophyllus* (accession PI 494573) and *H. petiolaris* (accession PI 435843). The subsequent generations were tested for BSR resistance in greenhouse trials. The BC_1_F_1_ resistant plants were used as male parents in a backcross to HA 89, and the selected BC_2_F_1_ resistant plants were advanced to the BC_2_F_2_ generation. The progenies of the resistant plants were self-pollinated and selected three times and advanced to the BC_2_F_5_ generation.

**Table 2:** **Sclerotinia basal stalk rot disease incidence in the recurrent parent, checks, and F_1_ plants derived from crosses with wild sunflower accessions of the *H. argophyllus* and *H. petiolaris***.

**Plant ID**	**Parents/checks/F_1_s**	**No. of plant tested**	**Disease incidence (%)**
10-122	Cargill 270 (S-check)	48	96.0
10-001	HA 89 (recurrent parent)	38	36.0
10-121	HA 441 (R-check)	48	14.0
10-137	Croplan 305 (R-check)	44	18.0
10-128	(NMS HA89 × *H. argophyllus* PI 494573)	22	4.5
10-124	(NMS HA89 × *H. petiolaris* ssp. *fallax* PI435815)	44	7.0
10-125	(NMS HA89 × *H. petiolaris* ssp. *fallax* PI435843)	21	5.0
10-126	(NMS HA89 × *H. petiolaris* PI451978)	44	11.0
10-127	(NMS HA89 × *H. petiolaris* ssp. *fallax* PI 468811)	44	2.0

### Greenhouse evaluations of sclerotinia BSR resistance in the BC_2_F_2_ populations

A greenhouse evaluation of the BC_2_F_2_ populations for resistance to BSR was performed in the winter of 2011 and the early spring of 2012. The recurrent parent HA 89, susceptible check Cargill 270, and two resistant checks, HA 441 and Croplan 305, were also included as controls in each set of tests. Nine BC_2_F_2_ populations of *H. argophyllus* were inoculated with the Sclerotinia isolate of NEB-274. The number of the tested plants in the populations ranged from 32 to 168 with a total number of 644 plants. As expected, the BC_2_F_2_ populations showed a wide variation of DI, ranging from 21.4 to 80.0% with a mean DI of 39.0% (Table 3). Two of the BC_2_F_2_ populations, 11–281 and 11–283, gave the lowest DI of 28.6 and 21.4%, respectively, which was similar to the resistant checks of HA 441 (27.8%) and Croplan 305 (25.0%).

**Table 3:** **Summary of the Sclerotinia basal stalk rot tests of the BC_2_F_2_populations in the greenhouse derived from crosses with wild sunflower accessions of the *H. argophyllus* and *H. petiolaris***.

**Line/Plant ID**	**Pedigree**	**No. of plants tested**	**No. of dead plants**	**Disease incidence (%)**
***H. argophyllus***				
Cargill 270 (S-check)		36	35	97.2
HA 89 (recurrent parent)		36	24	66.7
HA 441 (R-check)		36	10	27.8
Croplan 305 (R-check)		36	9	25.0
11-272	HA89//HA458/(NMS HA89 × *H. argophyllus* PI 494573)	46	37	80.0
11-273	HA89//HA458/(NMS HA89 × *H. argophyllus* PI 494573)	38	16	42.0
11-274	HA89//HA458/(NMS HA89 × *H. argophyllus* PI 494573)	48	24	50.0
11-275	HA89//HA458/(NMS HA89 × *H. argophyllus* PI 494573)	48	21	44.0
11-276	HA89//HA458/(NMS HA89 × *H. argophyllus* PI 494573)	32	16	50.0
11-280	HA89//HA458/(NMS HA89 × *H. argophyllus* PI 494573)	48	24	50.0
11-281	HA89//HA458/(NMS HA89 × *H. argophyllus* PI 494573)	168	48	28.6
11-282	HA89//HA458/(NMS HA89 × *H. argophyllus* PI 494573)	48	28	58.0
11-283	HA89//HA458/(NMS HA89 × *H. argophyllus* PI 494573)	168	36	21.4
Total		644	250	39.0
***H. petiolaris***				
Cargill 270 (S-check)		36	34	94.0
HA 89 (recurrent parent)		36	30	83.0
HA 441 (R-check)		36	16	44.4
Croplan 305 (R-check)		36	13	36.0
11-255	HA89//HA458/(NMS HA89 × *H. petiolaris* ssp. *fallax* PI435843)	192	138	71.9
11-256	HA89//HA458/(NMS HA89 × *H. petiolaris* ssp. *fallax* PI435843)	312	190	60.9
11-257	HA89//HA458/(NMS HA89 × *H. petiolaris* ssp. *fallax* PI435843)	48	35	73.0
11-258	HA89//HA458/(NMS HA89 × *H. petiolaris* ssp. *fallax* PI435843)	48	39	81.0
11-279	HA89//HA458/(NMS HA89 × *H. petiolaris* ssp. *fallax* PI435843)	168	119	70.8
Total		768	521	67.8

Higher disease incidences were observed in the five BC_2_F_2_ populations of *H. petiolaris;* although, an increased DI in the two resistant checks was also observed in this test (Table 3). The DI of the five populations ranged from 60.9 to 81.0% with a mean DI of 67.6% compared to 44.4 and 36.0% for the resistant checks HA 441 and Croplan 305, respectively.

### Field evaluation of BSR resistance of introgression lines

#### BC_2_F_3_ and BC_2_F_4_ evaluations

Twelve BC_2_F_3_ families of *H. argophyllus* were field tested at Carrington, ND and Crookston, MN in 2012, and another eight BC_2_F_3_ families were tested at Crookston, MN in 2013. The 2-year (2012 and 2013) mean DI of Cargill 270, HA 89, HA 441, and Croplan 305 was 47.4, 33.0, 31.9, and 19.9%, respectively, whereas all 20 of the *H. argophyllus* BC_2_F_3_ families had a DI lower than 20% (Table 4). Among these BC_2_F_3_ families, five had no infection, five had a DI lower than 10%, and the remaining 10 families had a DI lower than 20%. Among the 11 *H. petiolaris* BC_2_F_3_ families tested in 2012, four had a DI lower than 10%, two had a DI lower than 20%, while the remaining five had a DI that ranged from 29.5 to 54.6% (Table 4). The field-test results of the BC_2_F_3_ families showed a similar trend to the greenhouse evaluations of the BC_2_F_2_ population, where the *H. argophyllus* had a higher level of BSR resistance than the *H. petiolaris*. A total of eight plants from the *H. argophyllus* BC_2_F_3_ families, 11-275-037 and 11-283-037, and 18 plants from *H. petiolaris* BC_2_F_3_ families, 11-256-049 and 11-256-053, were selected based on their BSR DI and advanced to the BC_2_F_4_ generation.

**Table 4:** **Summary of the Sclerotinia basal stalk rot tests of the BC_2_F_3_ families in the inoculated field nurseries at Carrington and Crookston in 2012 and Crookston in 2013**.

**Line/Plant ID**	**Pedigree**	**No. of plants scored**	**Disease incidence (%)**
Cargill 270 (S-check)		141	47.4^ⱡ^
HA 89 (recurrent parent)		108	33.0^ⱡ^
HA 441 (R-check)		133	31.9^ⱡ^
Croplan 305 (R-check)		118	19.9^ⱡ^
11-273-001	HA89//HA458/(NMS HA89 × *H. argophyllus* PI 494573)	41	0.0
11-275-037	HA89//HA458/(NMS HA89 × *H. argophyllus* PI 494573)	53	0.0
11-283-017	HA89//HA458/(NMS HA89 × *H. argophyllus* PI 494573)	13^*^	0.0
11-283-037	HA89//HA458/(NMS HA89 × *H. argophyllus* PI 494573)	71	0.0
11-283-081	HA89//HA458/(NMS HA89 × *H. argophyllus* PI 494573)	13^*^	0.0
11-283-145	HA89//HA458/(NMS HA89 × *H. argophyllus* PI 494573)	38^*^	3.3
11-281-121	HA89//HA458/(NMS HA89 × *H. argophyllus* PI 494573)	29^*^	4.8
11-283-139	HA89//HA458/(NMS HA89 × *H. argophyllus* PI 494573)	35^*^	6.1
11-283-101	HA89//HA458/(NMS HA89 × *H. argophyllus* PI 494573)	37^*^	7.8
11-275-041	HA89//HA458/(NMS HA89 × *H. argophyllus* PI 494573)	57	8.9
11-282-013	HA89//HA458/(NMS HA89 × *H. argophyllus* PI 494573)	59	10.1
11-275-025	HA89//HA458/(NMS HA89 × *H. argophyllus* PI 494573)	55	10.3
11-275-017	HA89//HA458/(NMS HA89 × *H. argophyllus* PI 494573)	68	11.0
11-273-025	HA89//HA458/(NMS HA89 × *H. argophyllus* PI 494573)	52	11.3
11-283-041	HA89//HA458/(NMS HA89 × *H. argophyllus* PI 494573)	69	13.1
11-281-013	HA89//HA458/(NMS HA89 × *H. argophyllus* PI 494573)	72	13.2
11-281-141	HA89//HA458/(NMS HA89 × *H. argophyllus* PI 494573)	32^*^	13.9
11-282-017	HA89//HA458/(NMS HA89 × *H. argophyllus* PI 494573)	53	15.5
11-282-001	HA89//HA458/(NMS HA89 × *H. argophyllus* PI 494573)	61	15.9
11-283-080	HA89//HA458/(NMS HA89 × *H. argophyllus* PI 494573)	33^*^	16.7
11-256-049	HA89//HA458/(NMS HA89 × *H. petiolaris* ssp. *fallax* PI435843)	62	0.0
11-256-053	HA89//HA458/(NMS HA89 × *H. petiolaris* ssp. *fallax* PI435843)	68	0.0
11-279-017	HA89//HA458/(NMS HA89 × *H. petiolaris* ssp. *fallax* PI435843)	61	4.3
11-256-033	HA89//HA458/(NMS HA89 × *H. petiolaris* ssp. *fallax* PI435843)	67	8.5
11-257-025	HA89//HA458/(NMS HA89 × *H. petiolaris* ssp. *fallax* PI435843)	61	14.6
11-255-037	HA89//HA458/(NMS HA89 × *H. petiolaris* ssp. *fallax* PI435843)	47	15.6
11-255-025	HA89//HA458/(NMS HA89 × *H. petiolaris* ssp. *fallax* PI435843)	56	29.5
11-256-129	HA89//HA458/(NMS HA89 × *H. petiolaris* ssp. *fallax* PI435843)	63	30.2
11-256-029	HA89//HA458/(NMS HA89 × *H. petiolaris* ssp. *fallax* PI435843)	57	33.2
11-256-133	HA89//HA458/(NMS HA89 × *H. petiolaris* ssp. *fallax* PI435843)	64	43.5
11-255-129	HA89//HA458/(NMS HA89 × *H. petiolaris* ssp. *fallax* PI435843)	60	54.6

ⱡ *The disease incidence is the mean of 2 years of data*.

* *Tested in 2013 only*.

In 2013, the eight selected *H. argophyllus* and 18 *H. petiolaris* BC_2_F_4_ plants were evaluated for BSR resistance at Crookston, MN. The mean DI for Cargill 270, HA 89, HA 441, and Croplan 305 was 72.6, 51.6, 28.6, and 34.9%, respectively (Table 5). All eight *H. argophyllus* BC_2_F_4_ plants had lower DI scores than both of the resistant checks, ranging from 0 to 16.2%. Among the 18 *H. petiolaris* BC_2_F_4_ plants, all but one had a lower DI than the resistant checks, ranging from 4.0 to 25.4% (Table 5). A total of eight BC_2_F_4_ plants were selected and advanced to the BC_2_F_5_ generation.

**Table 5:** **Summary of the Sclerotinia basal stalk rot tests of the BC_2_F_4_ plants in the inoculated field nurseries at Crookston in 2013**.

**Lines/ Plant ID**	**Pedigree**	**No. of plants scored**	**Disease incidence (%)**
Cargill 270 (S-check)		77	72.6
HA 89 (recurrent parent)		56	51.6
HA 441 (R-check)		68	28.6
Croplan 305 (R-check)		57	34.9
12-3424-4	HA89//HA458/(NMS HA89 × *H. argophyllus* PI 494573)	30	0.0
12-3424-2	HA89//HA458/(NMS HA89 × *H. argophyllus* PI 494573)	28	4.1
12-3424-1	HA89//HA458/(NMS HA89 × *H. argophyllus* PI 494573)	31	7.1
12-3416-4	HA89//HA458/(NMS HA89 × *H. argophyllus* PI 494573)	86	9.3
12-3416-10	HA89//HA458/(NMS HA89 × *H. argophyllus* PI 494573)	44	9.8
12-3416-6	HA89//HA458/(NMS HA89 × *H. argophyllus* PI 494573)	85	10.4
12-3424-3	HA89//HA458/(NMS HA89 × *H. argophyllus* PI 494573)	27	11.7
12-3416-7	HA89//HA458/(NMS HA89 × *H. argophyllus* PI 494573)	82	16.2
12-3405-2	HA89//HA458/(NMS HA89 × *H. petiolaris* ssp. *fallax* PI435843)	80	4.0
12-3405-5	HA89//HA458/(NMS HA89 × *H. petiolaris* ssp. *fallax* PI435843)	81	4.0
12-3405-8	HA89//HA458/(NMS HA89 × *H. petiolaris* ssp. *fallax* PI435843)	69	4.0
12-3406-9	HA89//HA458/(NMS HA89 × *H. petiolaris* ssp. *fallax* PI435843)	42	4.1
12-3406-5	HA89//HA458/(NMS HA89 × *H. petiolaris* ssp. *fallax* PI435843)	74	5.6
12-3405-1	HA89//HA458/(NMS HA89 × *H. petiolaris* ssp. *fallax* PI435843)	72	7.4
12-3405-4	HA89//HA458/(NMS HA89 × *H. petiolaris* ssp. *fallax* PI435843)	65	8.0
12-3406-4	HA89//HA458/(NMS HA89 × *H. petiolaris* ssp. *fallax* PI435843)	77	9.0
12-3405-9	HA89//HA458/(NMS HA89 × *H. petiolaris* ssp. *fallax* PI435843)	42	10.9
12-3405-3	HA89//HA458/(NMS HA89 × *H. petiolaris* ssp. *fallax* PI435843)	77	12.5
12-3405-6	HA89//HA458/(NMS HA89 × *H. petiolaris* ssp. *fallax* PI435843)	87	14.3
12-3406-7	HA89//HA458/(NMS HA89 × *H. petiolaris* ssp. *fallax* PI435843)	74	20.1
12-3406-8	HA89//HA458/(NMS HA89 × *H. petiolaris* ssp. *fallax* PI435843)	48	20.6
12-3406-2	HA89//HA458/(NMS HA89 × *H. petiolaris* ssp. *fallax* PI435843)	82	22.8
12-3406-3	HA89//HA458/(NMS HA89 × *H. petiolaris* ssp. *fallax* PI435843)	70	23.7
12-3405-7	HA89//HA458/(NMS HA89 × *H. petiolaris* ssp. *fallax* PI435843)	84	23.8
12-3406-6	HA89//HA458/(NMS HA89 × *H. petiolaris* ssp. *fallax* PI435843)	83	25.4
12-3406-1	HA89//HA458/(NMS HA89 × *H. petiolaris* ssp. *fallax* PI435843)	85	30.9

### Eight promising BSR-resistant introgression lines

The selected eight promising BSR resistant lines were further evaluated for resistance to Sclerotinia BSR at Carrington and Grandin, ND in 2014 and 2015. The performance of the eight introgression lines for their reaction to BSR across seven environments is presented in Table 6. The DI varied across the years and/or locations. The lowest BSR DI was observed at the Grandin environment in 2015 (mean 1.9%), whereas the highest DI was observed at Crookston (mean 18.4%) in 2013 (Table 6). The introgression lines consistently exhibited high levels of BSR resistance across all of the environments. The mean BSR DI of the six *H. argophyllus* introgression lines ranged from 0.8 to 7.7%, and the two *H. petiolaris* introgression lines were 2.5 and 4.4%, while it was 36.1, 31.0, 19.5, and 11.6% for Cargill 270, HA 89, HA 441, and Croplan 305, respectively (Table 6).

**Table 6:** **Sclerotinia basal stalk rot tests of selected introgression lines derived from crosses with the wild sunflower species *H. argophyllus* and *H. petiolaris* at multiple locations of North Dakota and Minnesota from 2012 to 2015**.

**Line/Plant ID**	**Disease incidence (%)**							
	**Mean**	**2015 (BC**_**2**_**F**_**5**_**)**		**2014 (BC**_**2**_**F**_**4**_**)**		**2013 (BC_2_F_4_/F_3_)**	**2012 (BC**_**2**_**F**_**3**_**)**	
		**Grandin**	**Carrington**	**Grandin**	**Carrington**	**Crookston**	**Carrington**	**Crookston**
Cargill 270 (S-check)	36.1	10.0	17.6	34.6	37.4	72.6	45.0	24.6
HA 89 (recurrent parent)	31.0	4.9	18.6	31.8	39.5	51.6	22.3	25.0
HA 441 (R-check)	19.5	2.1	3.8	29.7	6.8	28.6	39.2	27.8
Croplan 305 (R-check)	11.6	2.1	1.9	11.2	7.9	34.9	14.7	10.0
H.arg 1/14-1562	3.0	0.0	0.0	2.5	3.3	9.3	0.0	0.0
H.arg 2/14-1563	3.2	0.0	0.0	7.2	4.2	4.8	NA	NA
H.arg 3/14-1565	0.8	0.0	0.0	2.8	0.0	0.0	NA	NA
H.arg 4/14-1568	7.2	2.1	4.4	10.5	9.1	0.0	0.0	0.0
H.arg 5/14-1570	7.7	0.0	10.0	13.4	4.6	6.1	NA	NA
H.arg 6/14-1573	1.9	0.0	0.0	3.7	0.9	3.3	NA	NA
H.pet 1/14-010	2.5	0.0	3.0	NA	NA	5.6	0.0	0.0
H.pet 2/14-1555	4.4	1.9	2.8	13.2	3.5	4.0	0.0	0.0
Mean	10.7	1.9	5.2	14.6	10.7	18.4	15.2	10.9
LSD (0.05)	7.8	4.3	11.6	13.0	9.2	16.1	13.2	14.9

*NA, not available*.

### GBS analysis of the eight promising introgression lines

The GBS protocol identified 29,644 SNPs, which were genotyped in the parents and introgression lines. A total of 8845 SNPs had either missing data in one parent or showed a polymorphism between HA 89 and HA 458, and another 1456 SNPs assigned to S18 (unknown LG) were removed, leaving a total of 10,498 SNP markers for further analysis. The SNPs identified from the GBS were not evenly distributed throughout the sunflower genome, with the lowest number in LG6 (236) and the highest in LG10 (1034; Table 7). Out of the total 10,498 filtered SNPs, only 462, 423, 275, 210, 216, and 188 SNPs were polymorphic with the cultivated sunflower parents in the introgression lines H.arg 1, H.arg 2, H.arg 3, H.arg 4, H.arg 5, and H.arg 6, respectively (data not shown). The number of polymorphic SNPs for the two *H. petiolaris* introgression lines, H.pet 1 and H.pet 2, was only 53 and 60, respectively (data not shown). Among the six *H. argophyllus* introgression lines, all but H.arg 6 detected a very high number of polymorphic SNPs on LG8 of the sunflower genome. On average, 19.4% of the SNP alleles on LG8 were recovered from BSR resistance donor parent in these five lines (Table 7, Table S1). These lines also retained a shared set of 117 SNP alleles (79.6% of the polymorphic SNPs), indicating common introgression regions on LG8 (Tables S1, S2). Most of these shared SNPs (87/117) were distributed between the 101 and 192 Mb region on the physical map of LG8 (Table 8). Only the introgression line H.arg 6 had a higher number of polymorphic SNPs, where 11.6% were detected on LG3. The introgression lines, H.arg 1 and H.arg 2, had additional *H. argophyllus* segments detected on the common regions of LGs 9 and 11. The highest number of polymorphic SNPs, 20.6 and 20.0%, were detected on LG11 of H.arg 1 and H.arg 2, respectively (Table 7, Tables S1, S2). Most of the shared SNPs of the two introgression lines were located in the regions between 201 and 250 Mb on LG9, and between 101 and 150 Mb on LG11 (Table 8). Additionally, 4.6, 7.0, and 7.2% polymorphic SNPs were also detected on LG10 in H.arg 1, H.arg 3, and H.arg 6, respectively. The latter two lines shared 49% of the polymorphic SNP alleles on LG10, while H.arg 1 shared 36.5% of polymorphic SNP alleles with H.arg 3 and H.arg 6 (Tables S1, S2). Out of the 51 shared SNPs on LG10, 46 (90.2%) were located between the 201 and 350 Mb region (Table 8). Overall, the introduced *H. argophyllus* segments in the six introgression lines were mainly recovered on LGs 3, 8, 9, 10, and 11 of the sunflower genome.

**Table 7:** **Tracking of the alien segments introduced from *H. argophyllus* and *H. petiolaris* in the highly basal stalk rot resistant introgression lines using single nucleotide polymorphism markers developed through a genotyping-by-sequencing approach**.

**Line**	**Percentage of polymorphism (%)**																
	**LG1 (594)**	**LG2 (532)**	**LG3 (630)**	**LG4 (470)**	**LG5 (921)**	**LG6 (236)**	**LG7 (324)**	**LG8 (697)**	**LG9 (796)**	**LG10 (1034)**	**LG11 (564)**	**LG12 (608)**	**LG13 (632)**	**LG14 (675)**	**LG15 (446)**	**LG16 (445)**	**LG17 (894)**
H.arg 1	1.2	2.3	2.9	1.3	1.4	0.4	0.9	20.2	6.0	4.6	20.6	3.0	0.6	0.9	1.1	2.2	0.7
H.arg 2	1.2	2.1	2.9	1.5	1.1	0.4	0.3	19.7	5.5	2.3	20.0	3.1	0.8	0.9	0.8	2.0	0.8
H.arg 3	1.0	1.1	1.3	0.2	1.6	0.0	0.0	18.8	0.4	7.0	0.2	2.3	0.6	0.4	0.0	0.7	0.9
H.arg 4	1.7	0.8	1.1	0.2	0.8	0.4	0.3	19.2	0.4	1.0	0.0	2.3	0.6	0.6	0.0	0.4	0.8
H.arg 5	1.2	0.6	1.1	0.2	0.9	1.7	0.0	19.1	0.4	2.2	0.0	1.8	0.3	0.3	1.3	0.7	0.3
H.arg 6	0.2	0.4	11.6	0.4	0.9	0.0	0.0	0.0	0.0	7.2	0.5	0.0	0.0	0.0	0.6	0.7	2.2
H.pet 1	0.0	0.2	0.6	0.0	0.0	0.4	0.0	4.3	0.0	0.0	0.5	2.0	0.5	0.1	0.2	0.0	0.2
H.pet 2	0.2	0.2	0.0	0.0	0.5	0.8	0.0	4.9	0.0	0.1	0.0	0.3	0.8	0.0	0.4	0.0	0.2

*The number of SNP markers is in parentheses*.

*The intensity of the green color indicates the proportion of the polymorphism between the parents and the introgressed lines*.

**Table 8:** **Distribution of the polymorphic SNP markers of H.arg 6 in LG3 and the shared SNPs of the introgression lines in LGs 8, 9, 10, and 11**.

**Line**	**Linkage group**		**Physical regions (Mb)**							**Total No. shared SNPs**
	**LG**	Length (Mb)^*^	**0–50**	**51–100**	**101–150**	**151–200**	**201–250**	**251–300**	**301–350**	
H.arg 6	3	203.5	18	31	18	6				73
H.arg 1 to 5	8	192.1	10	20	40	47				117
H.pet 1 and 2	8	192.1	3	4	17	4				28
H.arg 1 and 2	9	253.5	4	3	4	0	37			48
H.arg 1, 3, and 6	10	327.8	0	1	1	3	16	18	12	51
H.arg 1 and 2	11	208.7	26	27	43	10	4			111

* *The physical length of the linkage group was taken from http://sunflowergenome.org/genomeassembly.html*.

Unlike the *H. argophyllus* introgression lines, most of the polymorphic SNPs of the *H. petiolaris* introgression lines were detected only on LG8 (Table 7). The introgression lines H.pet 1 and H.pet 2 had 4.3 and 4.8% polymorphic SNPs, respectively, on LG8 and retained a shared set of 28 SNP alleles (77.8% of polymorphic SNPs; Table S3). Seventeen of the 28 shared SNPs were distributed to a region between 101 and 150 Mb on LG8 (Table 8).

### Detection of downy mildew resistance in the introgression lines

One of the parents, HA 458, used in this study carries the *Pl*_17_ downy mildew resistance gene (Qi et al., 2015). The eight introgression lines were first screened using the three DNA markers SFW04052, ORS963, and SFW08268, which are linked to *Pl*_*17*_ with an order SFW04052/*Pl*_*17*_/ORS963/SFW08268 at the position of 14.3/16.4/17.2/18.2 cM in the genetic map (Qi et al., 2015). Out of the eight introgression lines, five had the same PCR pattern at three marker loci (Table 9). Two recombination events were detected in the lines H.arg 1 and H.arg 4 between SFW04052 and ORS963, and another recombination occurred between ORS963 and SFW08268 in H.arg 3 (Table 9). SFW04052 was distal to ORS963 at 2.9 cM, whereas, SFW08268 was proximal to ORS963 at 1.0 cM in the genetic map (Qi et al., 2015). Thus, more recombination occurred between SFW04052 and ORS963 during the backcrossing and selection. The introgression lines were further inoculated with the downy mildew isolate of NA race 734, and the phenotypic data were consistent with marker data of ORS963 because ORS963 is the closest marker linked to *Pl*_17_ at a genetic distance of 0.8 cM (Qi et al., 2015). Two lines (H.arg 5 and H.pet 2) with an ORS963/*Pl*_*17*_ allele from HA 458 were homozygous resistant, and three lines (H.arg 1, H.arg 3, and H.arg 4) with a heterozygous allele were segregating, whereas three lines (H.arg 2, H.arg 6, and H.pet 1) with the HA 89 allele were homozygous susceptible (Table 9).

**Table 9:** **Results of the downy mildew and markers tests of the introgression lines**.

**Line**	**DM score**			**DNA markers for *Pl_17_***		
	**S**	**R**	**Phenotype**	**SFW04052**	**ORS963**	**SFW08268**
HA 89	15	0	S	A	A	A
HA 458	0	16	R	B	B	B
H.arg 1/14-1562	3	13	Seg.	**B**	**H**	H
H.arg 2/14-1563	15	0	S	A	A	A
H.arg 3/14-1565	7	10	Seg.	H	**H**	**A**
H.arg 4/14-1568	4	18	Seg.	**B**	**H**	H
H.arg 5/14-1570	0	16	R	B	B	B
H.arg 6/14-1573	18	0	S	A	A	A
H.pet 1/14-010	16	0	S	A	A	A
H.pet 2/14-1555	0	20	R	B	B	B

*S, susceptible; R, resistant; Seg., segregating; A, HA 89 PCR pattern; B, HA 458 PCR pattern; H, heterozygous*.

*The bold capital letters indicate recombination between markers*.

## Discussion

The wild *Helianthus* species are a valuable gene pool for sunflower genetic improvement for resistance to biotic and abiotic stresses. Currently, there are no cultivated sunflower inbred lines or commercial hybrids that possess an acceptable level of resistance to Sclerotinia (Hahn, 2002; Gulya, 2005; Talukder et al., 2014, 2016). In an effort to manage sunflower Sclerotinia disease, numerous wild *Helianthus* species were screened for their reaction to Sclerotinia head rot and stalk rot. High levels of resistance to Sclerotinia were reported in both wild annual and perennial sunflower species, as well as in their interspecific hybrids (for review see Seiler, 2010; Vear and Grezes-Besset, 2016). Block and Gulya (2008) and Block et al. (2009, 2010) tested BSR resistance in approximately 460 accessions from 14 wild *Helianthus* annual species and identified *H. argophyllus, H. debilis, H. praecox*, and *H. petiolaris* as potential sources of BSR resistance. In the present study, we successfully transferred Sclerotinia BSR resistance from the wild *Helianthus* annual species *H. argophyllus* and *H. petiolaris* into the cultivated sunflower. Eight alien introgression lines were selected from two crosses of HA 89 with *H. argophyllus* and *H. petiolaris* through seven disease-screening cycles (F_1_ to BC_2_F_5_) and showed stable resistance to Sclerotinia BSR across all environments in 4 years. The mean DI in the eight lines was significantly lower than those of the susceptible check Cargill 270 and the recurrent parent HA 89, as well as, the resistant check of the inbred line HA 441 (Table 6). The commercial hybrid check, Croplan 305 showed a good level of BSR resistance in almost every environment tested. However, in a given environment conducive to BSR incidence, even this resistant hybrid also suffered considerable damage as observed in the 2013 growing season. Notably, all of the introgression lines showed significantly lower disease in that season, suggesting that the introgressed resistance from the wild *Helianthus* species is more robust at minimizing BSR incidence in sunflower. As expected from a polygenically controlled quantitative trait, the introduced *H. argophyllus* alien segments in the cultivated sunflower were detected on LGs 3, 8, 9, 10, and 11 of the sunflower genome by GBS, whereas the introduced *H. petiolaris* alien segments were mostly detected on LG8 (Table 7). Because of the selection against BSR, these retained alien segments in the cultivated sunflower background are likely associated with BSR resistance. SNP markers within the introgression regions of *H. argophyllus* and *H. petiolaris* in the resistant lines are assumed to be good candidates for identifying segments carrying BSR-resistant QTL.

Due to multigenic nature of BSR resistance that relies on more than a few genes for optimal expression, a large number of progeny must be screened to access the effective selection of introgression in backcrossed populations and recover individuals that combine multiple alien transfers associated with BSR resistance. A greenhouse evaluation in early generations is necessary in order to reduce population size and focus on the selection of divergent pools with BSR resistance. The incubation temperature of the soil and the amount of inoculum are the critical factors in the greenhouse screening, and the best differentiation of resistance was obtained in the range of 21–24°C with 120 g of inoculated millets per tray. However, even in the strictly controlled greenhouse conditions in winter or early spring, the disease incidence of BSR across different tests showed a wide range of variation, especially for the resistant checks. For example, the resistant check HA 441 had a DI of 14.0, 27.8, and 44.4% in the three greenhouse tests, whereas the susceptible check Cargill 270 exhibited a stable DI (96, 97.2, and 94%) in the respective tests (Tables 2, 3). Selection pressure was much higher in the greenhouse than that in the field. For example, the mean DI of Cargill 270 in the field over 4 years was 36.1%, whereas the mean DI of Cargill 270 in the greenhouse was 95.7%. Thus, the selected progeny from the greenhouse tests retained a higher level of BSR resistance. In order to verify BSR resistance in the introgression lines in the present study, a large-scale field evaluation was used over 4 years. Combining the early generation greenhouse screening and field evaluation, the eight introgression lines were developed with higher levels of BSR resistance.

The use of NGS and GBS in wild germplasm has a profound affect because the SNP markers could be rapidly developed on a genome-wide scale and help to target the more narrowly defined genomic regions to trace introgression (Tiwari et al., 2014; Arbelaez et al., 2015; Winfield et al., 2016). In our initial screening of more than 500 sunflower SSR markers, no polymorphisms were detected in the resistant introgression lines of *H. petiolaris*, H.pet 1 and 2 (data not shown). A screening of H.pet 1 and 2, using GBS, discovered a total of 10,498 SNPs, which was over a 20-fold increase of marker density. Although a majority of the SNP markers had very low polymorphism levels across the genome, polymorphic SNP markers were detected on LG8 at 4.3 and 4.9% in H.pet 1 and 2, respectively (Table 7). The relatively low polymorphism between *H. petiolaris* with the cultivated sunflower might be attributed its recent origin, which is 0.75–1.0 million years divergent from *H. annuus*, resulting in the retention of large syntenic regions (Burke et al., 2004; Yatabe et al., 2007). Another possible reason is that the minor QTL associated with BSR resistance were lost under the high selection pressure in the early generations, resulting in the retention of only a few alien segments.

Unlike the *H. petiolaris* introgressions, relatively high polymorphisms were detected in the *H. argophyllus* introgression lines, suggesting that a considerable amount of diversity exists in *H. argophyllus*. Common introgression regions were detected among the introgression lines. For example, a common introgression region was observed on LG8 in all of the introgression lines except H.arg 6, two regions on LGs 9 and 11 between H.arg 1 and H.arg 2, and another region on LG10 among H.arg 1, H.arg 3, and H.arg 6 (Table 7, Table S1). The introgression region detected on LG3 in H. arg 6 is unique and might possess BSR resistant genes/QTL different from the other introgression lines.

Amouzadeh et al. (2013) reported QTL conferring partial resistance to BSR using a recombinant inbred line (RIL) population derived from a cross of PCA2/RHA 266. The five QTL for the percentage of necrotic area, based on controlled growth chamber tests, were located on LGs 1, 3, 8, 10, and 17 with the small effects, and each QTL explained between 0.5 and 3.2% of the observed phenotypic variance in the RIL population. Talukder et al. (2016) identified two major BSR resistance QTL on LGs 10 and 17 in multiple environments of a RIL population derived from a cross of HA 441/RHA 439, each explaining 31.6 and 20.2% of the observed phenotypic variance, respectively. An additional four QTL were also detected on LGs 4, 9, 11, and 16 in only one environment. Each of these QTL explains between 6.4 and 10.5% of the observed phenotypic variation. In the present study, the alien segments in the eight BSR resistant introgression lines were detected on LGs 3, 8, 9, 10, and 11, which are the same linkage groups where previously reported QTL were located. In addition to the BSR QTL, LG8 possesses a large *R* gene cluster harboring one rust (*R*_*1*_) and five downy mildew (*Pl*_*1*_, *Pl*_*2*_, *Pl*_*6*_, *Pl*_*7*_, and *Pl*_*15*_) resistance genes (Slabaugh et al., 2003; Yu et al., 2003; de Romano et al., 2010), and the largest number of nucleotide binding site and leucine-rich repeat (NBS-LRR) sequences, which encode proteins associated with disease resistance (Radwan et al., 2008). Notably, seven of the eight resistant introgression lines developed in this study had the alien segments detected in LG8, suggesting that new QTL of BSR resistance from the wild species are also present in this linkage group. The two QTL on LG10 detected by Amouzadeh et al. (2013) and Talukder et al. (2016) are located at a region between 253.4 and 281. 3 Mb, while ~34 polymorphic SNPs (66.7% of the shared SNPs) in LG10 that detected alien segments in three introgression lines were also located in this region (Table 8). In LG9, 77.1% of the shared SNPs were located within the 50 Mb region between 201 and 250 Mb, while the polymorphic SNPs in LG3 and the shared SNPs in LG11 were more widely spread in these two LGs (Table 8). Further QTL mapping will identify QTL regions associated with introgressed BSR resistance in LGs 3, 8, 9, 10, and 11. We have developed advance backcross QTL mapping populations using *H. argophyllus* and *H. petiolaris*. A genetic dissection of the target regions will elucidate the underlying genetic mechanism of BSR resistance in these wild species.

Sunflower downy mildew is another destructive disease globally. In the present study, an inbred line HA 458 harboring the *Pl*_*17*_ gene, which is resistant to all known *P. halstedii* races identified in the USA so far, was used as an elite parent in the transfer of BSR resistance from the wild species into a cultivated sunflower (Hulke et al., 2010; Qi et al., 2015; Gilley et al., 2016). In all of the generations, no selection was made against downy mildew. However, in the eight BSR resistant introgression lines of BC_2_F_5_, the marker screening and phenotypic test for resistance to downy mildew identified that five introgression lines exhibited resistance to downy mildew, and two were homozygous resistant, whereas three were heterozygous resistant (Table 9). The results indicated that the resistance from HA 458 was preferentially transmitted in the progenies. The germplasms combining resistance to Sclerotinia BSR and downy mildew represent a valuable genetic source for sunflower disease breeding.

## Ethics statement

The experiments were performed in compliance with the current laws of the USA.

## Author contributions

Conceived and designed the experiments: LQ. Performed the experiments: LQ, YL, ZT, GS, TG, CB. Analyzed data: LQ, YL, ZT. Wrote the paper: LQ. Commented on the manuscript before submission: ZT, GS.

### Conflict of interest statement

The authors declare that the research was conducted in the absence of any commercial or financial relationships that could be construed as a potential conflict of interest.
